# Synchronous brain networks for passive auditory perception in depressive states: A pilot study

**DOI:** 10.1016/j.heliyon.2019.e02092

**Published:** 2019-07-20

**Authors:** Kunihiro Aiba, Eri Miyauchi, Masahiro Kawasaki

**Affiliations:** Department of Intelligent Interaction Technology, Graduate School of Systems and Information Engineering, University of Tsukuba, 1-1-1, Tennodai, Tsukuba-shi, Ibaraki, 305-8573, Japan

**Keywords:** Psychology, Neuroscience, Synchronization, Depression, Default mode network, Time-frequency analysis, Electroencephalogram, Transfer entropy

## Abstract

Recent studies have revealed a strong relationship between the default mode network (DMN) and major depression disorder (MDD). The DMN consists of several areas in the brain where activity simultaneously increases during the resting state and is suppressed during cognitive tasks (i.e., DMN suppression). Although the DMN has been evaluated in patients with MDD, it has not been studied in people with self-measured depressive symptoms without medication. Although most studies have used high-demand cognitive tasks, the relationships between MDD and passive sensory tasks remain unclear. Here, we recorded electroencephalograph (EEG) data under two sessions: a resting session and an auditory session. Moreover, we assessed depressive states with a Self-Rating Depression Scale (SDS) score. To reveal the DMN suppression mechanism in the depressive states, we used EEG time-frequency analysis. As a result, the alpha-band phase synchronization in the DMN increased during the resting session and decreased during the auditory session. The results suggest that participants in a depressive state have both an abnormal DMN connectivity and a suppressed DMN connectivity via a passive stimulus. Moreover, we were able to estimate the DMN suppression mechanism during the depressive states: (1) the beta-band phase resetting was found in the auditory and parietal areas via the auditory stimulus; (2) the beta-band transfer entropy from the auditory area to the parietal area was high as information flow among these area; and (3) the beta-band systems (information flow) were synchronized with the alpha-band DMN systems. Although the sample size was small, these results suggest that the DMN systems may already be altered during self-measured depressive symptoms like the early stages of the depressive states.

## 1 Introduction

Recent studies have revealed a strong relationship between the default mode network (DMN) and major depression disorder (MDD). The DMN is defined as several regions that are simultaneously active during resting states (Raichle et al., 2001). It is known that the DMN is characterized by the function of self-reference during the resting state (Sheline et al., 2010). Through this function, many relationships between the DMN and several mental disorders have been revealed (Broyd et al., 2009). For example, the DMN abnormalities are reported to be seen in obsessive compulsive disorder (OCD) (Posner et al., 2017). Moreover, the DMN is connected to the amygdala, which is widely known to be related to MDD (Erk et al., 2010). Moreover, patients with MDD showed high connectivity between regions in the DMN (Hamilton et al., 2015; Demirtaş et al., 2016). Therefore, the DMN connectivity is one possible index of depressive states. However, although these studies focused on patients with MDD, few studies dealt with self-measured depressive symptoms. If we could identify the neural similarities between the self-measured depressive symptoms and the clinical diagnosed major depressions, the self-measured depressive symptoms would make opportunities to undergo the clinical assessments and treatments.

Several studies on the early symptoms of depression showed that the score dependent on stressful life events (Brown et al., 1973) was related to the risk of MDD (Kendler and Gardner, 2010). Moreover, it is known that cognitive therapy, one of the major treatments for MDD, only has favorable outcomes in patients with mild depression (Merrill et al., 2003). For these reasons, we need to be able to detect patients with depression at an early stage. Therefore, we focused on identifying the characteristics of DMN function in self-measured depressive symptoms as well as early (less severe) depressive states (Posner et al., 2016).

The DMN is involved in several cognitive functions. In comparison to the resting states, DMN connectivity decreased during working memory tasks (Greicius et al., 2003). DMN suppression is particular to high-demand cognitive tasks and not to simple passive sensory tasks (Greicius and Menon, 2004). In contrast, DMN suppression was not observed in patients with severe mental disorders, even during high-demanding cognitive tasks (Anticevic et al., 2012). Thus, in regard to passive stimuli tasks, we hypothesized that DMN connectivity in patients with MDD would be not suppressed.

There have been interesting findings on sensory thresholds and depressive moods. For example, in one study, depressive moods were related to lower sensory thresholds (Watanabe et al., 2005). According to Dunn's model of Sensory Processing, participants with a low sensory threshold would be in a state of sensory sensitivity for the passive stimuli task (Dunn, 1997; Brown et al., 2001). These results suggest that DMN connectivity in depressive states might be lower as DMN should be suppressed during cognitive processing.

If the passive stimulus could induce DMN suppression, it should be considered whether the stimulus actually caused the suppression. In other words, we must confirm the information flow from stimulus input to DMN suppression. To address these issues, an electroencephalograph (EEG) time-frequency analysis is useful. In previous studies, EEG analysis could evaluate the relationships between signals from different regions or different frequencies. Moreover, EEG has high time resolution, providing a time series analysis.

The present study focused on information flow in the network with transfer entropy (TE). TE calculates the causality between the two signals by using entropy and the time lag (Schreiber, 2000). For example, the information flow between the distant brain regions were identified by calculating the EEG phase reset with transcranial magnetic stimulation (TMS) (Kawasaki et al., 2014). In neuroscience, the Granger causality is another known causality estimation (Granger, 1969). However, it is not suitable in cases with model-free analyses as it requires pre-verified models (Vicente et al., 2011). Therefore, we applied TE as a model-free measurement in the causality analysis.

Moreover, we also attempted to evaluate the relationships between the stimuli-related area and DMN by quantifying the strength of its integration. We focused on the Phase synchronization index (PSI) as an integration measure of two systems. In a previous study, phase synchronization was crucial as a mechanism that integrates the incoming and endogenous activities, such as bottom-up and top-down (Varela et al., 2001). Phase synchronization represents the temporal coincidence of two signals (Varela et al., 2001). Previously, PSI was mainly used for quantifying the integration of two systems from different regions. However, we focused not only on the region, but also on the frequency bands as the two different systems for PSI. For example, in contrast to the beta frequency band in the sensory response (Wróbel, 2000), the most prominent frequency band of DMN activity was the alpha-band in several studies (Knyazev et al., 2011). Therefore, we used phase-phase cross frequency coupling (CFC) analysis and applied the PSI formula, which can identify the interactions between the different frequency EEG signals. A previous study showed that the CFC between theta and alpha phases of the frontal region were related to switching brain states (Akiyama et al., 2017). We detected not only the information flow at the same frequency, but also the interactions between different frequencies.

Therefore, our purpose was to detect whether 1) participants who have self-measured depressive symptoms have the same characteristics as MDD during the resting state; 2) the unique activity of DMN in passive sensory tasks exist; and 3) the mechanism of information flow can be identified with EEG-based analysis. We considered the differences between participants who have self-measured depressive symptoms and healthy controls by using a Self-Rating-Depression Scale (Zung, 1965); the index is scored by subjective questionnaires without medical confirmation.

## 2 Materials and methods

### 2.1 Participants

Twenty healthy volunteers (7 females and 13 males; mean age 21.3 ± 1.4 years, right-handed) participated in this EEG experiment. The participants have normal ability of hearing for the reports of subjective questionnaires. The informed consents were obtained from all participants before they participated in this study. The study was approved by the Faculty of Engineering, Information and Systems, Research Ethics Committee of the University of Tsukuba in accordance with the Declaration of Helsinki. As a limitation, we confirmed that all participants have never taken anti-depressants and had no suspicion of depression. The depressive tendencies of each participant were scored using the Self-Rating-Depression Scale (SDS) translated to Japanese by Fukuda et al. (Zung, 1965; Fukuda and Kobayashi, 1973). Participants answered the SDS before the EEG recording. Each participant was divided into two groups based on their SDS score: the depressive state group (DS) or the healthy control group (HC). Participants who scored above 45, which indicates a lightly depressive state in Japan, were in the DS group (Miki, 2002).

### 2.2 Procedure

This study consisted of two sessions: the resting session and the auditory session, sequentially. In each session, participants were asked to think about nothing and to keep their eyes closed for 3 ​min. All participants wore an eye mask and earphone in both sessions to minimize outside noise. In the auditory session, a piano-sound at the C-note (main frequency: about 261 ​Hz, length: 0.2 ​s) was presented as the auditory stimulus through the earphone; participants were instructed to ignore the sound. To create the natural (easy to ignore) sound, the sound was created by using the open-source software MuseScore 2.0 (MuseScore BVBA, Sint-Denijs-Westrem, Belgium). The sound intervals were at 1 ​s.

### 2.3 EEG recording

The participants’ EEG data was recorded from 62 scalp electrodes (Ag/AgCl) embedded in an electro-cap, which were placed on the international 10/10 system. The EEG data was recorded using SynAmp2 (Neuroscan, El Paso, TX, USA) and amplified using NeuroScan equipment (Neuroscan, EI Paso, TX, USA). A sampling rate was 1000 ​Hz. The EEG data were filtered in the bandpass range from 0.1 ​Hz to 30 ​Hz. The reference electrodes were placed on both the right and left mastoids.

### 2.4 EEG data pre-processing

We analyzed the EEG data with MATLAB (The MathWorks, Inc., Natick, Massachusetts, USA). First, to reject the task-irrelevant noises, we removed the EEG data from the first 30 ​s and the last 30 ​s of each session. Next, the EEG data were segmented into 2.0 ​s epochs (-1.0 ​s–1.0 s from the onset of auditory stimulus presentation) in the auditory sessions. The 2.0 ​s epochs were segmented in the resting state, in the same manner.

To decrease the volume conduction effect, we performed current source density (CSD) analysis. CSD analysis transforms the EEG data to the voltage value on the surface of the head by applying the spherical Laplace operator. We used CSD analysis with the recommended parameters of a previous study (Perrin et al., 1989).

### 2.5 DMN connectivity

To identify DMN coordination, we used the exact low-resolution electromagnetic tomography (eLORETA) software. The eLORETA software offers a linear inverse solution for EEG signals that have no localization error to detect deep sources (Pascual-Marqui et al., 2002). In this study, we calculated connectivity between the medial prefrontal cortex (mPFC) and the posterior cingulate cortex (PCC) as the main regions of the DMN. We used coordinates which were identified by a previous study (Harrison et al., 2008).

To compute DMN connectivity, as a connectivity index, we used phase synchronization. Phase synchronization measures the non-linear dependence of two signals. Phase synchronization value ranges from 0 to 1. We calculated each phase synchronization for theta (4–8 ​Hz), alpha1 (8–10 ​Hz), alpha2 (10–13 ​Hz), beta1 (13–16 ​Hz), beta2 (16–20 ​Hz), beta3 (20–24 ​Hz), and beta4 (24–30 ​Hz) frequency bands. In phase synchronization, the modulus of the complex valued coherency between the normalized Fourier transforms and was not depended on any amplitude information. It was also known that connectivity strength tends to change over the time of the sequence in the resting state (Chang and Glover, 2010). To remove this bias, we relied on the focused time and calculated the connectivity as a mean of the phase synchronization segmented at 200 ​ms in 100 ​ms interval for the resting state session data.

### 2.6 Wavelet analysis

To calculate the phases of each period, we used wavelet transformation (Tallon-Baudry et al., 1996). Morlet wavelets were used as a mother wavelet function due to the high time resolution. To detect the phase activity over the course of time, we calculated the phase at each time point in each epoch. The phase was calculated as the arctangent of the results of the convolution of the measured EEG signal s(t) with a complex Morlet wavelet function w (t,f):

(1) $$w\left(t,f\right)=\sqrt{f}\exp\left(-\frac{t^{2}}{2{\text{σ}}_{t}^{2}}\right)\exp\left(i2\pi ft\right)$$

with ${\text{σ}}_{f}$ = 1/(2π${\text{σ}}_{t}$). In the wavelet calculation, we used the constant ratio (f/${\text{σ}}_{f}$= 7), with f ranging from 7 ​Hz to 30 ​Hz (1 ​Hz steps). Not to be affected by the previous or following stimuli, we used a small ratio (for Hz = 4, 5, 6, we used f/${\text{σ}}_{f}$ = 4,5,6 each) for each session.

### 2.7 Phase locking factor

To calculate the phase resetting by auditory stimulus, we used phase locking factors (PLF) ^32)^. PLF was calculated at each electrode (ch), time point (t), and frequency (f) as follows:

(2) $$PLF\left(t,f,ch\right)=\frac{1}{N}\overset{N}{\underset{n=1}{\sum }}\exp(i\varphi \left(t,f,ch,n\right))$$

where φ is the instantaneous phase of the wavelet transformed EEG and N is the total number of epochs. To reduce the sampling bias from the number of epochs, we calculated a standardized PLF (PLFz) by using an averaged baseline PLF (PLFb; PLF of internal auditory stimulus (500–900 ​ms from onset)):

(3) $$PLF_{z}\left(t,f,ch\right)=\frac{PLF\left(t,f,ch\right)-\bar{PLF_{b}\left(t,f,ch\right)}}{\sigma (PLF_{b}\left(t,f,ch\right))}$$

Moreover, we defined the PLFz peak as a section with the mean of all target electrode PLFz (mPLFz) higher than the 95% confidence interval calculated using the overall time of mPLFz in each participant. Specifically, to consider the statistical significance of the differences of the PLFz, we calculated the PLFz peak.

A previous study showed that the EEG alpha component correlated to DMN activity exists in the parietal area (Knyazev et al., 2011). Therefore, we calculated the PLFz of the parietal electrodes (Pz, P1, P2, P3, P4) as a response of the DMN and those of the temporal electrodes (FT7, T7, TP7, FT8, T8, TP8) as a response of the auditory area.

### 2.8 Transfer entropy

To confirm the existence of information flow, we calculated the TE (Schreiber, 2000). TE can estimate the causality between two signals by using the future state of X and the current states of X and Y.

We calculated the entropy rate (h_1_) and the entropy rate (h_2_) to use TE. These were represented as follows:

(4) $$h_{1}=-\underset{x_{t+\tau },\;x_{t},y_{t}}{\sum }p\left(x_{t+\tau },\;x_{t},y_{t}\right)\log_{2}p\left(x_{t+\tau }\text{|}x_{t},y_{t}\right)$$

(5) $$h_{2}=-\underset{x_{t+\tau },\;x_{t},y_{t}}{\sum }p\left(x_{t+\tau },\;x_{t},y_{t}\right)\log_{2}p\left(x_{t+\tau }\text{|}x_{t}\right)$$

where p (x|y) is the conditional probability and p (x, y) is the joint probability of system X and Y. The value of x_t_ and y_t_ are the current observation values of system X and Y, and x_t + τ_ is the time-shifted (shifted by τ) observation value of system X. Moreover, to compute h_2_, we assumed that the two systems are independent. Under this assumption, we could assume that the time-lagged observation value (x_t + τ_) of system X is also independent of the current observation value of the other electrode, y_t_.

The TE from system Y to X is defined with h_1_ and h_2_, as follows:

(6) $$TE_{Y\to X}=h_{2}-h_{1}=\underset{x_{t+\tau },\;x_{t},y_{t}}{\sum }p\left(x_{t+\tau },\;x_{t},y_{t}\right)\log_{2}\left(\frac{p\left((x_{t+\tau }|x_{t},y_{t}\right)}{p\left(x_{t+\tau }|x_{t}\right)}\right)$$

We can therefore estimate the directional information flow among the two systems as the formula is asymmetrical.

To calculate TE, we estimated the probability density. In a previous study, multi-dimensional histograms were constructed from the data (Schreiber, 2000). However, the method includes a bias in cases with small sample sizes; in other words, it is too sparse to estimate the probability density. Since our data had a very small sample size, we used the kernel density estimation method to estimate multi-dimensional probability density functions (Silverman, 1981). Furthermore, to focus on the phase oscillation, we used a von Mises distribution as a kernel density function. Because von Mises distribution is a probability distribution function on the circle, we could estimate the probability density continuously (i.e., p(x) = p (x+2π)).

Next, to compute TE, we calculated the estimated multi-dimensional probability density function. We estimated the joint probability at an arbitrary point, ($\tilde{x_{t+\tau }},\;\;\tilde{x_{t}},\;\;\tilde{y_{t}}$), as follows:

(7) $$p\left(\tilde{x_{t+\tau }},\tilde{x_{t}},\tilde{y_{t}}\right)\approx \frac{1}{P}\overset{P}{\underset{j=1}{\sum }}K\left(\tilde{x_{t+\tau }}-x_{t+\tau ,j},\text{κ}\right)K\left(\tilde{x_{t}}-x_{t,j},\text{κ}\right)K\left(\tilde{y_{t}}-y_{t,j},\text{κ}\right)$$

with K(θ,κ) as a von Mises distribution:

(8) $$K\left(\text{θ},\text{κ}\right)=\frac{\text{exp}\left(\text{κ}\;\text{cos}\left(\text{θ}\right)\right)}{2\pi I_{0}\left(\text{κ}\right)}$$

where$I_{0}\left(・\right)$ represents the modified Bessel function of the first kind and order 0, and κ is the concentration parameter that is the same in all three dimensions.

Moreover, we calculated the mean of all the participant's Root Mean Square Error (RMSE) as follows.

(9) $$RMSE\left(\tilde{x_{t+0}},\tilde{x_{t}},\tilde{y_{t}}\right)=\underset{x_{t+\tau },\;x_{t},y_{t}}{\sum }\sqrt{\left({\left(\text{p}\left(\tilde{x_{t+0}},\tilde{x_{t}},\tilde{y_{t}}\right)-\text{p}\left(\tilde{x_{t}},\tilde{y_{t}}\right)\right)}^{2}+{\left(\text{p}\left(\tilde{x_{t+0}},\tilde{x_{t}}\right)-\text{p}\left(\tilde{x_{t}}\right)\right)}^{2}\right)}$$

The RMSE represents the differences in estimated multi-dimensional probability density functions at τ = 0. Since TE should be theoretically 0 at τ = 0, RMSE should be as small as possible. We calculated RMSE ranging from κ = 1 to 20 in 0.5 step for each participant and then selected κ (= 11) as a kernel bandwidth which minimumizes the RMSE.

In TE estimations, the phase data was segmented in a 200 ​ms period around the time of auditory stimuli (pre-50 ​ms, post-150 ​ms), including the PLFz peak (Fig. 3), as the values of x_t_, y_t_. The phase data was also segmented from a time-lagged 200 ​ms period as the corresponding observation values x_t+τ_. We computed TE ranging from the time lag (τ) from 0 to 200 ​ms, in 10 ​ms steps, for each participant. The resting state TE was calculated by using phase data extracted in a continuous 200 ​ms period and a time-lagged 200 ​ms from the period around the random point of each epoch.

We tested whether the mean $TE_{X\to Y}$ around the stimuli was higher (or smaller) than the upper (or lower) limit of the 95% confidence interval of $TE_{Y\to X}$ distributions. We also tested the TE of the resting state.

### 2.9 Cross frequency coupling

To consider the relationship between different frequency oscillations, we calculated the phase–phase cross-frequency coupling (CFC) between two different oscillations at one electrode. We used the formula from Kawasaki et al. (Kawasaki et al., 2010) and applied the Phase Synchronization Index, using Δ$\Phi_{f_{1}f_{2}}$(t, j) as the phase difference between two fixed phases (α×$\text{Φ}\;(\text{of}\;f_{1}$Hz) – β×Φ (of $f_{2}$Hz)) at the *j*^*th*^ electrodes. Where, for the calculation of CFC, both $\Phi_{f_{1}}$ and $\Phi_{f_{2}}$ were multiplied by the appropriate parameters α and β. These showed α×$f_{1}$ and β×$f_{2}$ to be the least common multiple (LCM) of $f_{1}$ and $f_{2}$. The relationships between the two phases were expressed in the ratio “LCM/α: LCM/β” during the stimulus periods. The CFC was calculated with the following equation:

(10) $$CFC\left(i,j,f_{1},f_{2}\right)=\sqrt{{\left(\frac{\sum_{x=1}^{N}\;\cos\left(\Delta \Phi_{f_{1}f_{2}}\left(x,j\right)\right)}{N}\right)}^{2}+{\left(\frac{\sum_{x=1}^{N}\;\sin\left(\Delta \Phi_{f_{1}f_{2}}\left(x,j\right)\right)}{N}\right)}^{2}}$$

where Δ$\Phi_{f_{1}f_{2}}\left(x,j\right)$ is the phase difference at the *j*^*th*^ electrodes, and the number of time points N with an interval of 1 ​s is 200.

Furthermore, we computed a standardized CFC (CFCz) by using the averaged baseline CFC (CFCb; CFC of internal auditory stimulus (500–900 ​ms from onset)):

(11) $$CFC_{z}\left(x,j,f_{1},f_{2}\right)=\frac{CFC\left(x,j,f_{1},f_{2}\right)-\bar{CFC_{b}\left(x,j,f_{1},f_{2}\right)}}{\sigma (CFC_{b}\left(x,j,f_{1},f_{2}\right))}$$

Here, we used trial averaged CFC to calculate the CFCz.

### 2.10 Statistical analysis

In our study, the DS group only comprised of 4 participants, which is a very small sample size (DS = 4, HC = 16). In general, due to such a small sample size, the results of the statistical analyses may often introduce errors. In such cases, the Student's t-test is feasible if the effect size is large (over 0.8) (De Winter, 2013). Therefore, we used the Student's t-test, because the statistical differences in the data had a large effect size in this study. We used Cohen's d (Cd) as the effect size measurement.

## 3 Results

### 3.1 DMN connectivity

To estimate the DMN connectivity, we calculated the phase synchronization.

**Fig. 1:**
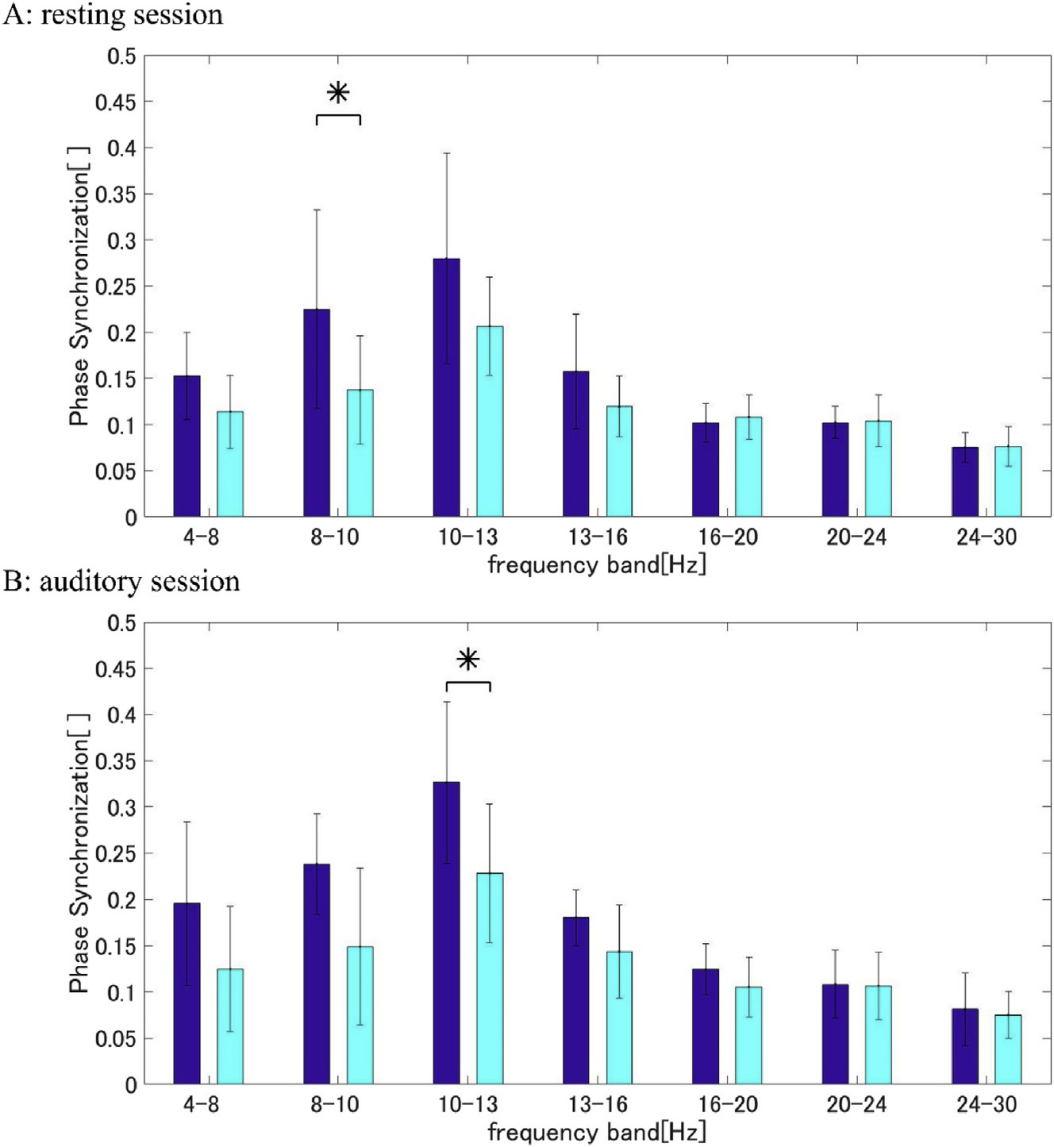
Group-averaged phase synchronization (blue: DS group, cyan: HC group). A: Resting session (mean of epoch); B: Auditory session (mean of 0–200 ​ms from the stimulus onset) (*P < 0.05; two-tailed t-test).

In the resting state session, the alpha bands phase synchronization was the highest of all frequency bands. There were significant differences in the synchronization between the DS and HC groups in the low alpha frequency band (two-tailed t-test: p = 0.036, Cd = 1.27) (see Fig. 1A).

In the auditory session, maximum phase synchronization was observed in the high alpha-band, which was also shown in the resting state sessions. Specifically, at the stimulus onset (0–200 ​ms from stimulus onset), phase synchronization of the DS group was significantly higher than in the HC group in the high alpha frequency band (two-tailed t-test: p = 0.036, Cd = 1.27) (see Fig. 1B).

**Fig. 2:**
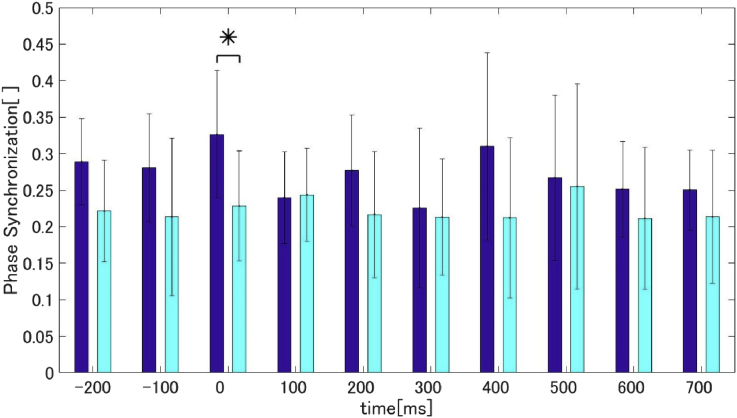
Time-course phase synchronization in auditory sessions (blue: DS group, cyan: HC group). The time in the figure shows calculation onset (window = 200 ​ms) (*P < 0.05; two-tailed t-test).

During the time-course phase synchronization of the high alpha-band in the auditory sessions, there were no significant differences between the DS and HC groups at 0 ​ms (calculated with 200 ​ms time window) (see Fig. 2). Phase synchronization of the DS group was seen to decrease after the onset of the stimulus (i.e., at 100 ​ms).

### 3.2 Phase locking factor

**Fig. 3:**
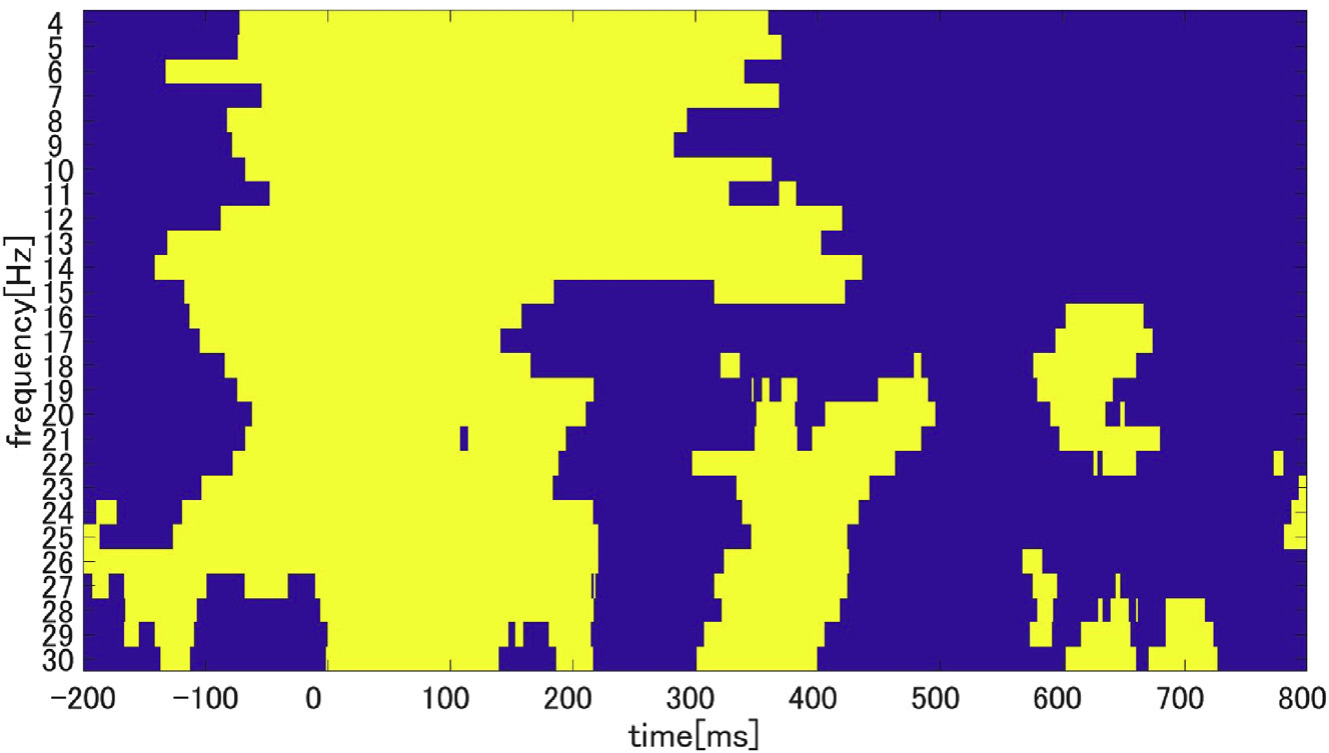
Participant-averaged (N = 20) time-frequency relationships of the PLFz peak (A: mean of auditory area, B: mean of parietal area). The yellow part indicates the PLFz peak which is the section where mPLFz is higher than the 95% confidence interval in each participant.

To detect the DMN suppression, we calculated PLFz. PLFz results showed peak of phase resetting that was induced by the auditory stimuli ranging from 4 to 30 ​Hz (Fig. 3). These resets were also observed before the auditory stimuli onsets due to the wavelet time resolution. For the response of the DMN, we focused on the time 0–100 ​ms from onset at the parietal area as the PLFz peak was found in all frequency bands (Fig. 3).

To identify the target electrodes that showed different responses between the two groups in the parietal areas, we compared the mean value of the PLFz peak (0–100 ​ms from onset) in some parietal electrodes (Pz, P1, P2, P3, and P4). We selected the activity of 30 ​Hz of the Pz electrode from some electrodes that had significant differences, since the Pz electrode showed the minimum p-value (see Table 1).

**Table 1:** Group-averaged PLFz of electrodes and frequency pairs which are significantly different among the two groups in the parietal area (*P < 0.05, **P < 0.01; two-tailed t-test).

frequency [Hz] – electrode [ch] pair	PLFz of depressive state (mean±std)	PLFz of helthy control (mean±std)	p-value (t-test)	Cohen's d
5 – P4	6.44±1.31	1.62±3.19	9.17$\times 10^{-3}$ **	1.63
26 – Pz	1.34±1.64	-2.57$\times 10^{-2}\pm 0.822$	2.59$\times 10^{-2}$ *	1.36
27 – Pz	1.64±1.86	-6.02$\times 10^{-2}\pm$0.935	1.58$\times 10^{-2}$ *	1.49
28 – Pz	1.72±1.36	-4.78$\times 10^{-2}\pm$1.11	1.35$\times 10^{-2}$ *	1.53
29 – Pz	1.85±0.984	-4.49$\times 10^{-2}\pm$1.22	1.05$\times 10^{-2}$ *	1.60
30 – Pz	2.04±1.05	-3.51$\times 10^{-2}\pm$1.22	5.94$\times 10^{-3}$ **	1.74

**Fig. 4:**
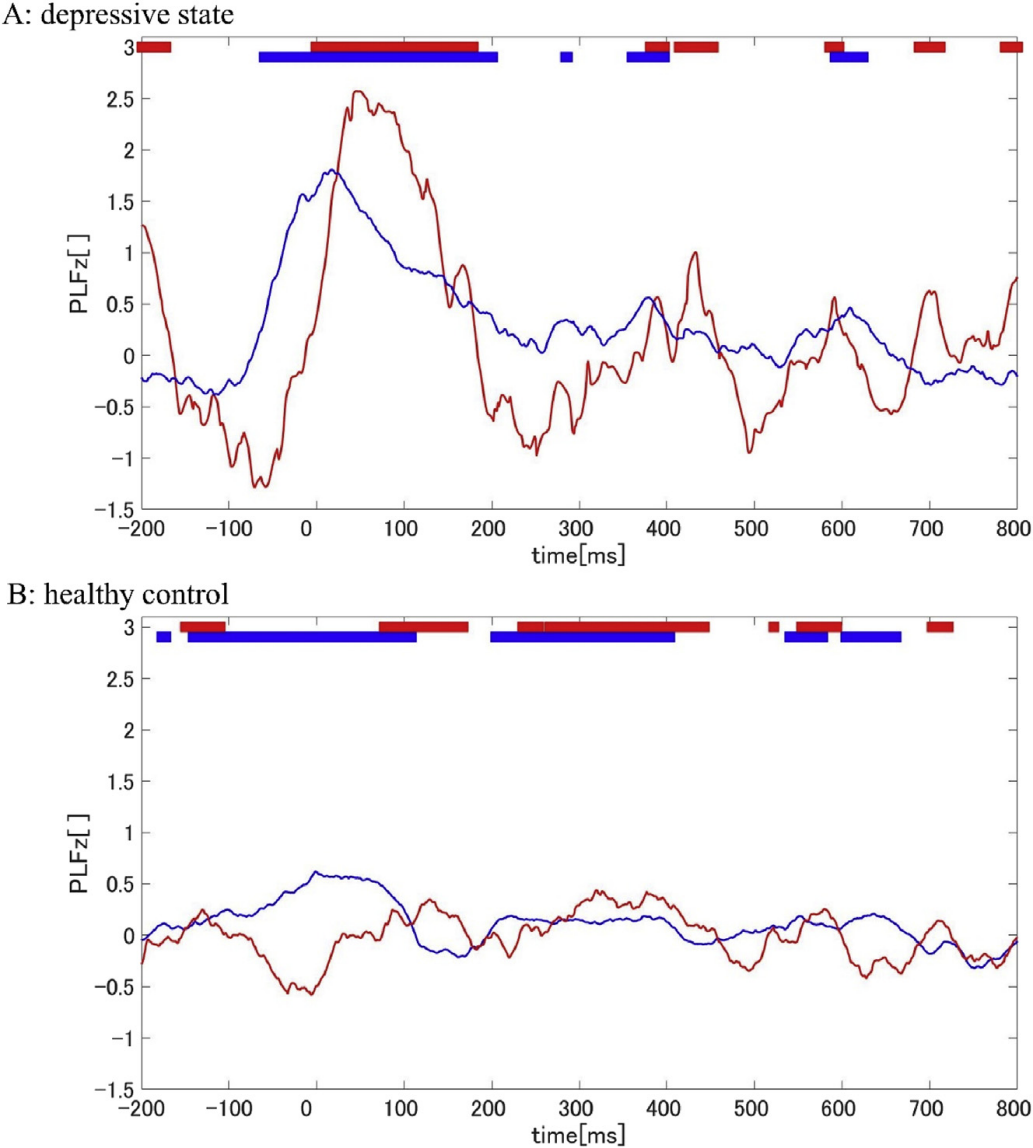
Time-course PLFz of Pz (polygonal blue line) and mean of auditory area (polygonal red line) in each group (A: DS group, B: HC group). The thick lines at the top of the graph indicate the time periods of the PLFz peak which were higher than the 95% confidence interval calculated by using the all-time PLFz of Pz electrodes (blue) and the auditory area (red) (P < 0.05).

To consider the relationship between the Pz and the auditory areas, we observed the time-lag of the phase reset between these areas (mean PLFz of FT7, T7, TP7, FT8, T8, and TP8) at 30 ​Hz (Fig. 4A, B). In the DS group, the high phase reset was found around the stimulus onset.

### 3.3 Transfer entropy

**Fig. 5:**
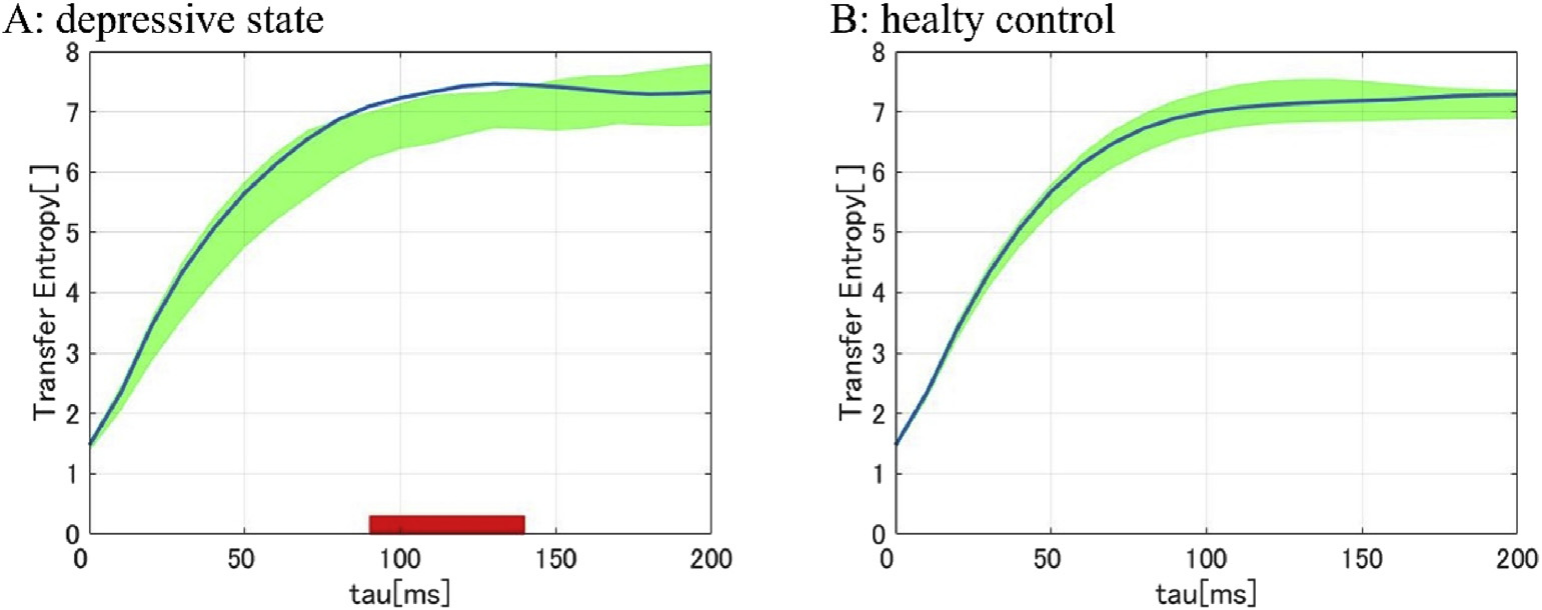
Transfer Entropy between Pz and FT7 in each group (A: DS group, B: HC group). Mean ${\text{TE}}_{FT7\to Pz}$ of each group shown as thick blue lines. Greenish areas indicate the 95% confidence intervals of ${\text{TE}}_{Pz\to FT7}$. The thick red lines in the lower part of the line graphs indicate the time periods in which ${\text{TE}}_{FT7\to Pz}$ were significantly higher than the ${\text{TE}}_{Pz\to FT7}$ (P < 0.05 with Bonferroni correction).

To determine whether the time-lag of the phase reset had causality, we calculated the TE between the Pz and each temporal electrode (FT7, T7, TP7, FT8, T8, TP8) from -50 ​ms (start of the auditory phase reset peak section) with a time window of 200 ​ms as the length of the peak section). We used the instantaneous phases of the CSD signals from two electrodes (Pz and each temporal electrode) as observation values of two systems. There was a significant difference in the TE in only the Pz and FT7 pair in the DS group. The ${\text{TE}}_{FT7\to Pz}$ is significantly higher than ${\text{TE}}_{Pz\to FT7}$ at tau = 90–140 ​ms. The minimum p-value was 0.0018 at tau = 130 ​ms (two-tailed t-test: Cd = 3.76, with Bonferroni correction) (Fig. 5A, B).

### 3.4 Cross frequency coupling

**Fig. 6:**
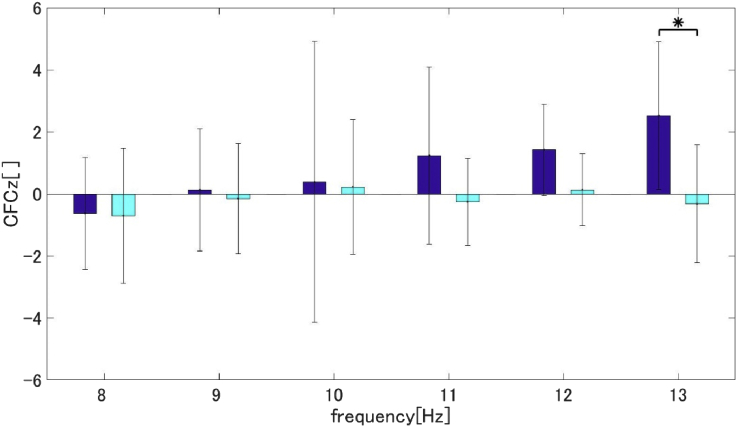
Group-averaged CFCz between 30 ​Hz and X Hz at the Pz electrode (blue: DS group, cyan: HC group). X is the frequency ranging from 8 to 13 ​Hz (*P < 0.05; two-tailed t-test, with no correction).

To examine the relationship between the system of information flow (30 ​Hz) and the DMN system (alpha-band), we calculated the CFCz. Based on the estimated time by TE, we calculated the CFC from 80 ​ms with a 200 ​ms time window (after 130 ​ms (most significant τ) from -50 ​ms (onset of TE calculation)). As a result, the CFCz between 13 ​Hz and 30 ​Hz showed significant differences between the two groups (two-tailed t-test: p = 0.020, Cd = 1.43) (Fig. 6).

**Fig. 7:**
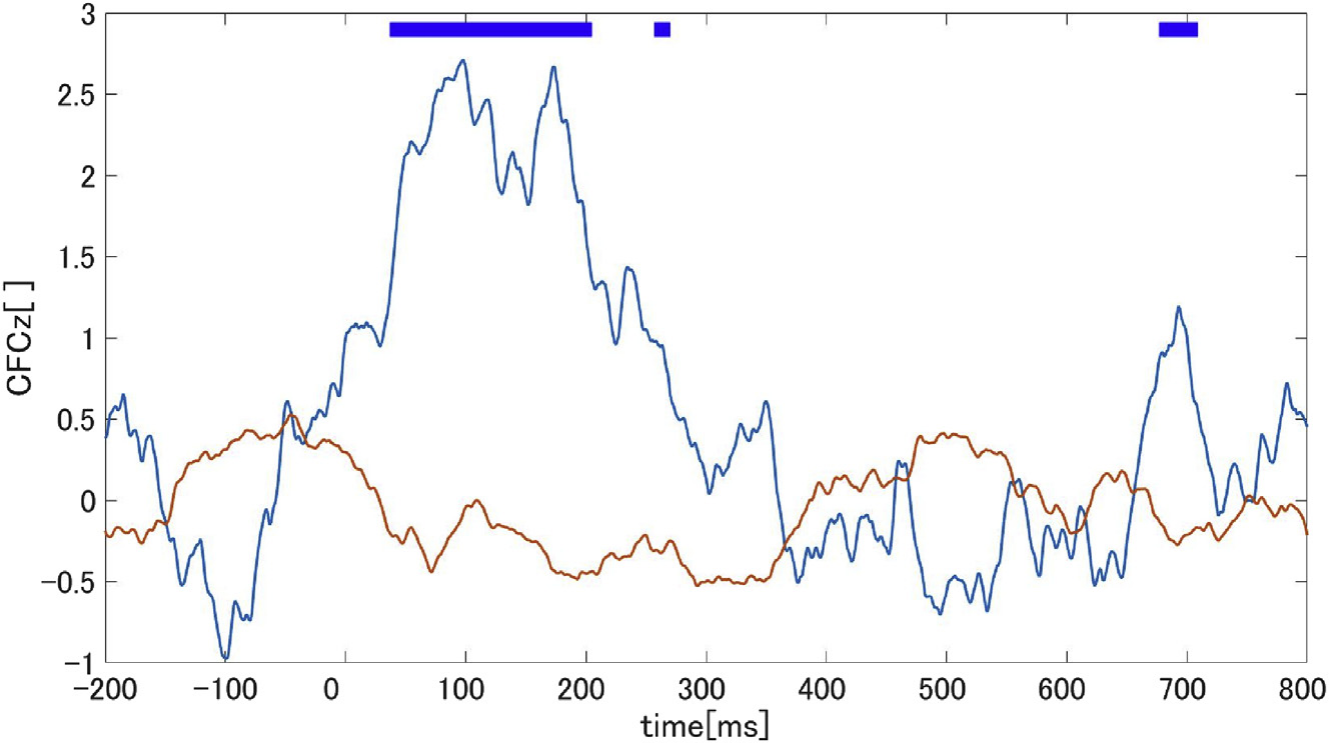
Time-course CFCz between 13 ​Hz and 30 ​Hz (polygonal blue line: DS group, polygonal red line: HC group). The thick blue lines at the top of the figures indicate the time periods which had significantly different CFCz among the two groups (i.e., P < 0.05).

In the time sequence of CFCz between 13-30 ​Hz, the CFCz of the DS group increased after the information transferred period estimated by the TE (Fig. 7).

## 4 Discussion

Our study showed that the hierarchal brain network in the depressive state could be detected by estimating the time-course relationships between the systems of different frequency bands in the high time-resolution EEG. We revealed two important findings. First, we detected that people in a depressive state without feeling subjective symptoms had high DMN connectivity, which is the same as seen in MDD. Next, we could analyze the mechanisms of DMN suppression in the passive stimulus task.

We identified three steps in the DMN suppression mechanism of the DS group in the process from stimulus onset to suppression. In the first step, the 30 ​Hz phase resetting was induced by an auditory stimulus. After the phase resetting, the causal analysis revealed the existence of information flow from the auditory areas to the parietal areas. Finally, both the cross-frequency analysis and DMN connectivity results indicated increased CFC between 13 ​Hz and 30 ​Hz on the parietal areas, which showed the suppression of DMN connectivity in the alpha-band. These results were identified by using high time-resolution EEG, classifying signal function by the frequency bands, and concerning the time-course activity.

The time-course DMN connectivity result possibly suggests that the DMN of the depressive states was suppressed by perceiving stimulus passively. There have been some studies about DMN suppression, depression, and the passive stimulus task. A previous study showed DMN suppression correlated to cognitive demand (Greicius et al., 2003). Here, the sensitivity of the stimulus in the passive sensory task was defined as having a high sensory threshold in the Dunn's Model of Sensory Processing (Dunn, 1997). One study showed that the thermal sensory threshold was higher in the depressive state (Watanabe et al., 2005). According to these studies, if we proposed that the amount of DMN suppression was a response to a stimulus, our results could imply that a high sensory threshold of a depressive state was shown for the auditory sensor. This relationship might lead us to think that the inverse response would occur whether perceiving a stimulus passively or actively. Therefore, we should investigate the passive response for another sensory stimulus in a future study.

The strength of the DMN connectivity in each of the frequency band results indicated that the main feature was in the alpha-band with DMN connectivity different in each of the participant's mental state. Our results could support a previous study that found that DMN connectivity is higher in the alpha-band (Qin et al., 2010). The relationship between the DMN parietal activity and the alpha frequency band has been considered in many studies using not only an EEG, but also through functional Magnetic Resonance Imaging (fMRI) (Chen et al., 2008; Jann et al., 2009). Therefore, we can definitively conclude that patients with a depressive state have high DMN connectivity in a resting state. Moreover, as our result are similar to other studies on MDD in the resting state, our study would be the first study to indicate that DMN connectivity is already altered before patients feel the subjective symptoms of depression.

The PLFz and TE results may imply that participants in a depressive state are easily affected by a passive stimulus and tend to transfer the information to the parietal area in a beta-band. The PLFz results showed significant differences in the phase reset at Pz between the two groups. Moreover, in the auditory area, a high phase reset occurred before the reset in the parietal area. In general, it could be thought that these phase resets were possibly effected by volume conduction (Tallon-Baudry et al., 1996). However, this effect should not be a concern since our results had a time duration and the CSD method was applied.

To determine the possibility of phase resetting in certain areas, we estimated the causal relationships. We identified significant information flow from the auditory area (FT7) to the parietal area (Pz) by considering the information transfer. A previous study conclude a causality between the two areas since the transfer time calculated by TE match the duration time of the phase reset (Kawasaki et al., 2014). This study is known as a demonstration of the hypothesis that activity-dependent synaptic modifications may drive the change in direction of information flow by observing the lag-phase (Żochowski and Dzakpasu, 2004; Olejarczyk et al., 2017). Following the method of previous study, we could demonstrate the hypothesis that a causality exists between the two areas which have the duration of the phase reset. Moreover, the prediction time estimated by the TE is nearly equal to the duration time of the phase reset. This means a causal relationship between the two areas. Therefore, this indicates that information of the auditory stimulus would transfer to the parietal area in participants with depressive states.

The correlation between intensity of transcranial magnetic stimulation (TMS) and phase resetting was revealed in a previous study (Kawasaki et al., 2014). Furthermore, the method could clarify the deficit of information flow from the visual areas to the motor areas in the patient with major depressions (Miyauchi et al., 2019). However, our results show that the auditory stimulus that participants could ignore induce a high phase reset (Fig. 4a). This result shows that participants in a depressive state had a characteristic relationship between the intensity of a stimulus and phase resetting. Moreover, this result may support the hypothesis that participants in a depressive state have a high sensory threshold (Watanabe et al., 2005).

Furthermore, it is possible that the CFCz results suggest that the transferred auditory information (in the beta-band) suppresses DMN connectivity (in the alpha-band). CFC is an index that quantifies the interaction between different frequency bands. We calculated the CFCz to investigate the relationship between the 30 ​Hz phase reset at Pz and the high alpha band, which was the main frequency band of the DMN. As a result, CFCz between 13 and 30 ​Hz was highest in the DS group at the transferred time estimated by the TE. This result was particularly observed in participants in a depressive state. In addition, we could confirm that CFCz increases during 50 ms–200 ​ms from onset only in the DS group. Previous studies showed that the EEG alpha component, which was correlated to DMN activity, existed at the parietal area (Knyazev et al., 2011; Laufs et al., 2003). Moreover, some studies showed parietal alpha oscillation activity decreased when task-related, which was also shown in DMN activity (Knyazev et al., 2011; Mo et al., 2013). These results imply that parietal alpha oscillation represent DMN activity. Therefore, our CFCz results are thought of as an integration of the two systems with DMN activity and beta-band systems (information flow) from auditory areas.

Here, a previous study showed an inverse correlation between EEG alpha or beta oscillation in a motor task and Blood Oxygenation Level Dependent (BOLD) technique in the sensorimotor cortex (Yuan et al., 2010). The authors concluded that the BOLD increases in the sensorimotor cortex and suppressed parietal alpha oscillation due to the time-course activity and estimated source coordinates. Our DMN connectivity results in the auditory sessions that showed significant differences at 0–200 ​ms. However, there were no significant differences at other time points. However, we did find that DMN connectivity of the DS group was decreased at 100–300 ​ms (Fig. 2). Therefore, it was thought that the DMN was suppressed as a result of an integration of the two systems (DMN activity and information flow).

As a limitation, our participants had not received the medical confirmation and only subjectively felt depressive symptoms. Since our study focused on patients with a depressive state, we did not do an additional study to compare them with patients with MDD. Future studies should be conducted that include patients with MDD. Moreover, due to the small sample number of participants, we were careful in our analysis, for example, concerning effect sizes. Therefore, it is noted that this is a pilot study. In future studies, a larger number of participants should be included.

## 5 Conclusions

In this study, we demonstrated that not only do participants in a depressive state exhibit the same DMN features in resting state as patients with MDD but that the mechanisms of DMN suppression to an auditory stimulus could be revealed by EEG time-frequency analysis. We recommend examination of the information flow from other sensory modalities in future studies. Furthermore, our results are important as DMN connectivity has already begun to alter before patients feel any symptoms. Our results would support the early detection of patients with self-measured depressive symptoms. As people perceive various kinds of stimulus passively, a study on depressive states might be very effective in revealing the causes of MDD or the mechanism that lead to its increasing severity. Because the number of studies performed on depressive states are limited, we must continue to examine them further to reveal their features.

## Declarations

### Author contribution statement

Kunihiro Aiba: Conceived and designed the experiments; Performed the experiments; Analysed and interpreted the data; Contributed reagents, materials, analysis tools or data; Wrote the paper.

Eri Miyauchi, Masahiro Kawasaki: Conceived and designed the experiments; Analyzed and interpreted the data; Contributed reagents, materials, analysis tools or data; Wrote the paper.

### Funding statement

The research was supported by Grant-in-Aid for Scientific Research on Innovative areas (15H01576), Grant-in-Aid for Scientific Research (C) (18K07587), and Program to Disseminate Tenure Tracking System MEXT.

### Competing interest statement

The authors declare no conflict of interest.

### Additional information

No additional information is available for this paper.
